# Surveillance of Q Fever in Dairy Cattle in Latvia: Molecular and Serological Findings and Association with Farm and Woodland Density †

**DOI:** 10.3390/microorganisms14040881

**Published:** 2026-04-14

**Authors:** Guntis Boikmanis, Didzis Elferts, Žanete Šteingolde, Artjoms Mališevs, Juris Ķibilds, Lelde Grantiņa-Ieviņa, Aivars Bērziņš, Olga Valciņa

**Affiliations:** Institute of Food Safety, Animal Health and Environment “BIOR”, 3 Lejupes Str., LV-1076 Riga, Latvia; guntis.boikmanis@bior.lv (G.B.); didzis.elferts@bior.lv (D.E.); zanete.steingolde@bior.lv (Ž.Š.); artjoms.malisevs@bior.lv (A.M.); juris.kibilds@bior.lv (J.Ķ.); aivars.berzins@bior.lv (A.B.); olga.valcina@bior.lv (O.V.)

**Keywords:** *Coxiella burnetii*, Q fever, epidemiology, spatial distribution

## Abstract

Q fever, caused by the obligate intracellular bacterium *Coxiella burnetii*, is a widespread zoonotic disease and a notifiable condition in 25 European Union countries. Dairy cattle are important reservoirs, and infection can pose a risk to both animal and human health. The aim of this study was to assess the temporal and spatial prevalence of *C. burnetii* in dairy farms in Latvia. In the period of January 2016–December 2018, seroprevalence was estimated. Between January 2022 and September 2023, bulk tank milk and pooled milk samples were collected from 5.81% of Latvian dairy farms and analyzed for the presence of *C. burnetii* DNA using PCR. Overall, 15.46% of milk samples and 10.05% of farms tested positive. These two periods were retrospectively compared with previously published Q-fever prevalence data within Latvian territory. To identify potential risk factors, statistical analyses were performed, including correlation assessments with farm-level and environmental variables. These new surveillance data and historical temporal spatial changes in disease distribution confirm the continued circulation of *C. burnetii* in Latvian dairy herds and highlight environmental and herd-level associated factors that may influence its spread. Results indicated that *C. burnetii* prevalence was positively associated with a high cattle density and overflowing plains, while the presence of surrounding woodlands was negatively correlated, suggesting a potential natural barrier effect.

## 1. Introduction

Q fever or Coxiellosis is a widespread zoonotic disease caused by the bacterium *Coxiella burnetii*. *C. burnetii* is a pleomorphic Gram-negative obligate intracellular bacterium known for its highly infectious nature and its strong resistance to external environmental stress [1]. It spreads in a spore-like cell form and infects mononuclear phagocytes and alveolar macrophages [2]. Q fever is a worldwide zoonotic disease, but the epidemiological manifestations differ regionally; therefore, it is included in the list of mandatory notifiable diseases in the European Union [3]. The largest outbreak of Q fever in a human population occurred in the Netherlands, with 4026 human cases reported [4]. Q fever can be asymptomatic or present as acute or chronic illness in humans, posing significant risks, particularly to individuals in close contact with infected livestock. Infected animals are usually chronically infected without symptoms of Q fever, but the disease causes abortions in ruminants [5]. There is substantial evidence that Coxiellosis plays a role in dairy cattle health issues such as abortion, stillbirth, perinatal mortality, weak calves, retained placenta, and infertility or sub-fertility [6]. The global molecular prevalence of *C. burnetii* at the herd level in cattle is estimated at 32.3% (95% CI: 25.3–40.01%), showing considerable variability across the included studies [7]. The neighboring country of Latvia, Estonia, had detectable *C. burnetii* exposure in humans and livestock, and dairy herds appeared to be among the higher-risk livestock groups in national surveys [8].

Because goat and cattle stables are not fully enclosed, bacteria from inside can spread to the surrounding outdoor areas. *C. burnetii* has been found in outdoor air samples near affected farms, across various particle sizes [9,10]. The bacterium and its spore-like forms are capable of surviving in the outdoor environment for weeks or even months [11]. The spread of Q fever is influenced by several factors, including animal density, rainfall, temperature, terrain features, vegetation, and wind speed [12]. Herd size was identified as a risk factor in multiple studies from Ireland, Denmark [13], the Netherlands [14], Portugal [15], and Italy [16]. In Italy, herds with more than 92 animals had a significantly increased risk [16], while in Denmark, herds exceeding 80 cows had 17.9-times-higher odds of testing positive than smaller herds [17]. Regional cattle density was associated with a higher risk in Sweden [12], France [18], and Denmark [17], with densities of 100–120 cows per km^2^ linked to a higher infection risk compared to ≤60 cows per km^2^. Wind-borne spread was reported as a more likely transmission route than animal trade [19], with strong winds and low precipitation recognized as additional risk factors in Sweden [12]. Risk zones were defined within a 5 km radius [20], though *C. burnetii* can disperse up to 30 km under strong winds [21]. Vegetated areas, such as forests, reduce wind speeds and promote dust particle settling due to canopy friction, suggesting a lower Q-fever prevalence in forested regions [22]. Other geographical and climatic factors influencing disease spread include the prevalence of arable land, peaty soils, groundwater levels, and average wind speed [20]. Open landscapes—such as areas near water bodies (including designated swimming areas), fields, clearcuts, and other spaces lacking tree cover—facilitate disease spread by promoting windier conditions compared to more enclosed environments like forests or mixed forest–field systems [19]. Since 2016, the literature has consistently supported the importance of airborne (wind-driven) transmission of *C. burnetii* from infected ruminants. Dry, open landscapes and high wind speeds are associated with increased transmission risk, whereas dense vegetation, forests, or artificial windbreaks can mitigate this risk by reducing dust and aerosol generation and enhancing particle deposition. Wind-driven airborne dispersal is now recognized as a dominant pathway for both community-level and between-herd transmission, with infectious dust capable of traveling several kilometers based on observational and modeling evidence [23]. Spatial analyses and systematic syntheses of outbreak data indicate that airborne dispersal can extend up to approximately 18 km under extreme wind conditions, with the highest human infection risk occurring within about 5 km of infected ruminant sources. These studies identify wind speed and direction, along with open landscape structure, as key determinants of spread [23]. Environmental conditions further modulate transmission risk: dry weather, low precipitation, and events generating large amounts of contaminated material—such as kid-ding or abortion in livestock and manure spreading—can elevate environmental *C. burnetii* levels and increase outbreak likelihood [24]. Air sampling in a post-outbreak region found *C. burnetii* DNA in ambient air, spatial variation was associated with the distance to/size of infected farms and temporal variation was linked to kidding seasons [24]. In a recent publication, scientists reviewed environmental sampling studies (dust, air, soil) and concluded that dust samples give the highest loads; evidence linked environmental positivity to livestock populations, but spatio-temporal dispersion evidence was considered to still be limited [25]. In a recent study in Spain, scientists concluded that *C. burnetii* DNA was detected in the air and/or dust of 80% of the livestock farms studied (20 out of 25 farms). This indicated a high prevalence of *C. burnetii* infection within the domestic cycle. The presence of *C. burnetii* DNA in aerosols was associated with abortions, while its presence in dust was significantly linked to dairy herds, which showed a higher risk compared to meat-producing herds. This study highlighted the importance of infection within the domestic cycle and the potential for environmental contamination [26]. 

Previous studies in Latvia have concluded that animal import and the territorial density of cattle and their herds, as well as their proximity to a positive farm, are important risk factors for the spread of Q fever [27,28]. A previous study in Latvia has also shown that wind speed is a significant risk factor in the spread of Q fever [28].

The aim of this study was to conduct passive and active surveillance of Q fever in Latvian dairy cattle farms during 2016–2018 and 2022–2023 using blood samples and bulk tank milk samples, respectively, and to compare the findings with historical molecular and serological data from 2012 onward. The study objectives were to investigate the temporal and spatial prevalence of *C. burnetii* in dairy herds and associations with environmental factors. Specifically, the influence of cattle density, as well as woodland coverage, on the occurrence of Q fever was assessed.

## 2. Materials and Methods

### 2.1. Study Design

The present study includes two periods: January 2016–December 2018 when seroprevalence was estimated, and January 2022–September 2023 when bulk tank milk and pooled milk samples were tested for a *C. burnetii* DNA presence. The study was a countrywide passive and active surveillance study to estimate the occurrence of Q fever, and therefore prevalence was obtained from a cross-sectional study of blood and milk samples.

For the retrospective analysis of Q-fever prevalence, previously published data were included from the periods of 2012–2015 and 2019–2023 [27,28,29,30].

### 2.2. Serological Tests of Blood Samples

In total, 1924 dairy cow blood samples from cattle that suffered abortion (passive surveillance), representing 737 dairy cattle sheds, were sampled during the three-year period of January 2016–December 2018. The samples were collected from cases of cattle abortions under the National Dairy Cattle Abortion Surveillance Programme.

All blood samples were screened for the presence of antibodies against *C. burnetii* by ELISA, using the ID Screen Q Fever Indirect Multi-species kit, and the results for serum samples were interpreted according to the manual provided by the manufacturer (IDvet, Grabels, France).

### 2.3. Milk Samples

For the active surveillance, in total, 1066 bulk tank or pooled milk samples from 657 dairy herds across Latvia during January 2022–September 2023 were collected as part of the annual National Animal Infectious Disease Surveillance Plans for compulsory brucellosis and leukosis testing. Each year, animals older than 24 months are tested for bovine brucellosis in 0.2% of cattle holdings, randomly selected by the Food and Veterinary Service in accordance with Cabinet of Ministers Regulations No. 881 (2012). To maintain an officially disease-free status for bovine enzootic leukosis, all holdings test animals once they reach 24 months, ensuring that at least 20% of officially free holdings with eligible animals are examined annually. The Food and Veterinary Service determines which holdings undergo mandatory testing in accordance with Cabinet of Ministers Regulations No. 880 (2011).

In total, milk samples from 657 dairy cattle sheds were examined, accounting for 5.11% of all dairy cattle sheds nationwide, of which there were 12,858 at the end of 2023 according to the data from the Agricultural Data Centre of Latvia (https://registri.ldc.gov.lv/, accessed on 27 November 2025). The sheds included in the study represented 31 local region in Latvia.

The milk samples were pooled by sampling personnel (veterinarian or farm owner), with each pool containing material from up to 50 animals. Milk samples (50 mL) preserved with 2-bromo-2-nitropropane-1,3-diol were transported to the laboratory in a cold box at 4–6 °C and were either processed immediately or stored at the same temperature for one to seven days before testing or aliquoting.

### 2.4. Molecular Tests of Milk Samples

Aliquoted 2 mL milk samples were stored in a −20 °C freezer. DNA was extracted from 200 µL of the bulk tank milk or combined milk samples using a QIAmp DNA mini kit (Qiagen, Hilden, Germany) or Dneasy mericon Food Kit (Qiagen, Hilden, Germany) according to the manufacturer’s protocols. Real-time PCR amplification of the *C. burnetii* target gene DNA *IS1111* and GAPDH, as an internal control of extraction and amplification steps, was carried out using the ADIAVET COX REAL TIME reagent kit (Bio-X Diagnostics, Rochefort, France). Part of the samples were tested using the ID Gene Q Fever Triplex kit or ID Gene Q Fever-Chlamydophila spp. Triplex (Innovative Diagnostics, Grabels, France). The reason for changing the reagents was solely related to procurement procedures. Internal positive and negative controls were used for all kits. The real-time PCR amplification was implemented using Rotor-Gene (Qiagen, Hilden, Germany), ViiA 7 (Thermo Fisher Scientific, Waltham, MA, USA) and QuantStudio 6 (Thermo Fisher Scientific, Waltham, MA, USA) real-time PCR cyclers. According to the manufacturer, the PCR detection limit of the ADIAVET COX REAL TIME reagent kit is 1.5 *C. burnetii* per 5 μL of DNA, and the quantification limit is 2 *C. burnetii* genome equivalents or bacteria per 5 μL. ID Gene kits amplify target genes and an endogenous internal control. Detection limits of these kits are DL_PCR_ < 10 copies/PCR, and specificity is 100%.

The DNA extraction date and real-time PCR results were compiled in an Excel spreadsheet.

### 2.5. Data Statistical Analysis

Datasets of the analyzed animals and sheds were retrieved from the Laboratory Information System.

The 95% confidence interval for disease prevalence was calculated with the WilsonCL algorithm [31] using the open-access program Epitools (https://epitools.ausvet.com.au/trueprevalence, accessed on 27 November 2025).

Newly obtained data were compared with historical data obtained by molecular and serological methods in Latvia starting from 2012 published previously [27,28]. Serological results represent *C. burnetii* antibodies in blood serum of cows that suffered abortions [27,28].

Information on the number of herds and animals in the districts was obtained from the free-access statistics of the Agricultural Data Centre of Latvia (https://registri.ldc.gov.lv/, accessed on 27 November 2025). Woodland type and density data were obtained from freely available statistical data of the State Forest Service of Latvia (https://www.vmd.gov.lv/lv/meza-statistikas-cd, accessed on 27 November 2025). Maps were created using QGIS 3.10.14-A Coruna (QGIS, Grüt (Gossau ZH), Switzerland).

Binary logistic regression was performed to assess the influence of the explanatory variables (woodland density indicators and cattle density) on the probability of Q fever. Before data analysis, all woodland density indicators were expressed as percentages of the municipality’s total area in order to answer the question of what change in the probability of Q fever corresponds to an increase of 1 percentage point in the proportion. Cattle density was expressed as the number per km^2^. Data analysis was conducted using R version 4.5.2 [32]. Pearson correlation analysis was performed on all explanatory variables to assess correlations among them, not including variables with high multicollinearity in the models. Subsequently, binary logistic regression was performed. In the first approach, a separate model was fitted for each explanatory variable, whereas in the second approach, two-variable models were constructed, which included cattle density and each other variable. The response variable was defined as the ratio between positive and negative tested herds. Results were expressed as odds ratios with 95% confidence intervals.

## 3. Results

### 3.1. Seroprevalence in the Period of 2016–2018

**Figure 1:**
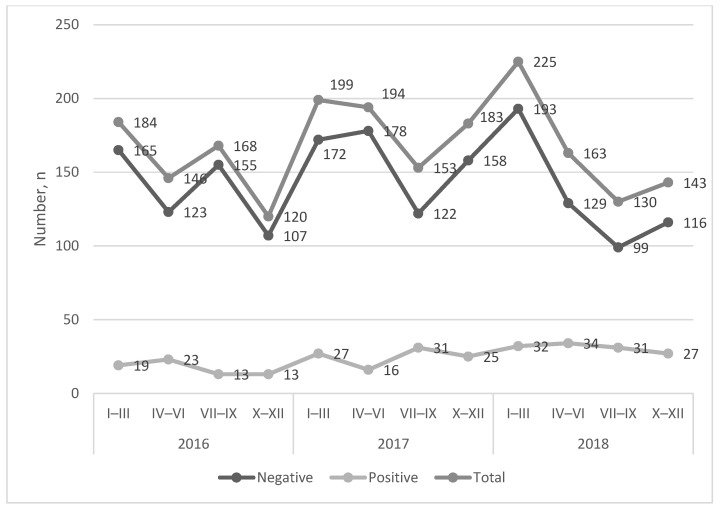
Seroprevalence in the period of 2016–2018. The graph is showing the total quarterly numbers of cattle that suffered abortions, as well as *C. burnetii* seronegative and seropositive samples.

**Figure 2:**
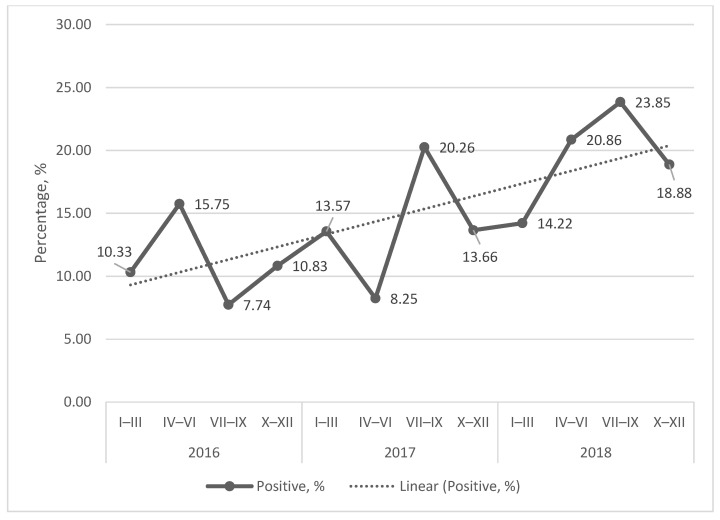
Seroprevalence in the period of 2016–2018. The graph is showing the quarterly percentage of *C. burnetii* seropositive cattle that suffered abortions and the linear trendline.

In the period of January 2016–December 2018, the *C. burnetii* seroprevalence of cattle that suffered abortions was 13.29% (95% confidence interval (CI): 0.1188–0.1485). In total, there were 268 *C. burnetii* seropositive samples from 2016 tested blood samples, representing 72 infected dairy cattle sheds. Additionally, 27 samples were evaluated as suspicious. The quarterly number of the cattle that suffered abortions was 167 on average. The number of *C. burnetii* seropositive samples did not show any trends of increase (Figure 1), but the percentage of them showed an increasing trend (Figure 2). The highest incidence peaks were observed in the second or third quarter of the year.

In the Western part of the country, the highest number of positive sheds was in Tukums, Saldus and Dobele counties. In the northeastern part of the country, the highest number of positive sheds was in Ogre and Cesis counties, while in the eastern part of the country, it was in Preili, Rezekne and Augsdaugava counties (Figure 5B).

### 3.2. Shedding of C. burnetii DNA in Milk

The findings for the period of 2022–2023 revealed that 169 milk samples of healthy cows, or 15.85% (95% confidence interval (CI): 0.1378–0.1817) of the total, tested positive for the presence of *C. burnetii* DNA. Furthermore, the bacterium was identified in 66 out of 657 sheds, spanning 21 counties, which accounts for 58% of the country’s total counties. In total, 10.05% of farms had at least one positive sample. Compared to the serological prevalence in 2016–2018, Q-fever infection was detected in a large number of sheds in the north of Latvia—Valmiera and Smiltene counties.

**Figure 3:**
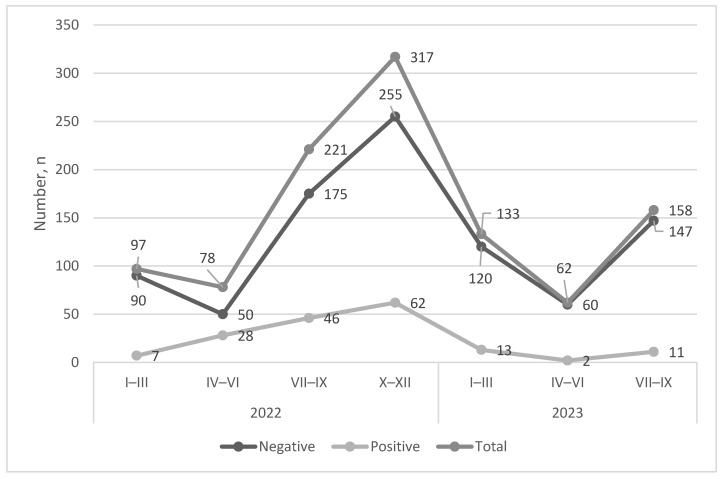
Shedding of *C. burnetii* DNA in milk, 2022–2023. The graph is showing the total quarterly numbers of milk samples, as well as *C. burnetii* DNA-negative and -positive samples of healthy cows.

**Figure 4:**
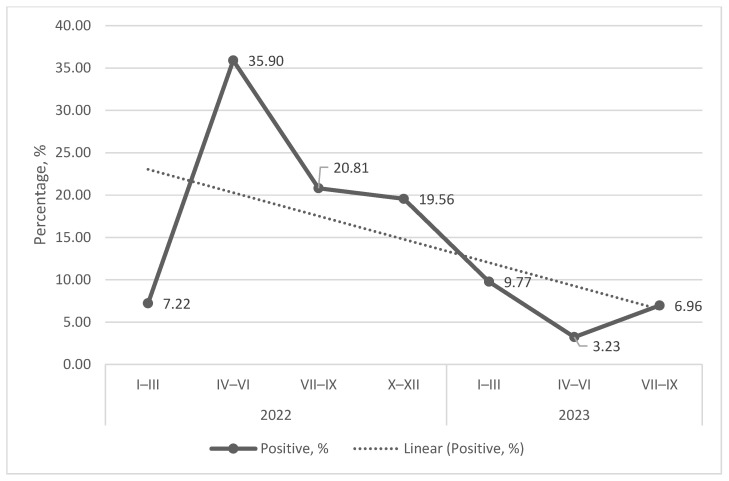
Shedding of *C. burnetii* DNA in milk, 2022–2023. The graph is showing the quarterly percentage of *C. burnetii* DNA-positive samples of healthy cows and the linear trendline.

The highest number of positive milk samples was detected in the fourth quarter of 2022, but this was also the highest number of milk samples tested. The highest percentage of milk samples was in the second quarter of 2022 (Figure 3). The overall trend for positive milk samples during the 2022–2023 period was to decrease (Figure 4).

### 3.3. Changes in the Prevalence of Q Fever over Time in Dairy Cattle in Different Regions

**Figure 5:**
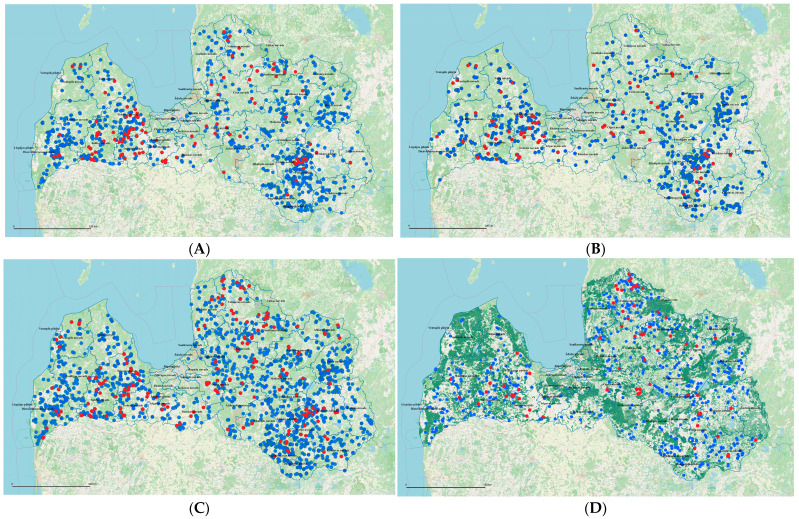
Changes in the prevalence of Q fever over time in dairy cattle in different regions of Latvia. (**A**)—Prevalence of Q fever in Latvia in 2012–2015 according to the serological results—*Coxiella burnetii* antibodies in blood serum of cows that suffered abortions (the original data have been published in [28]). (**B**)—Prevalence of Q fever in Latvia in 2016–2018 according to the serological results—*C. burnetii* antibodies in blood sera of cows that suffered abortions (the original data were obtained within the present study). (**C**)—Prevalence of Q fever in Latvia in 2019–2021 in milk samples (the original data have been published in [27]). (**D**)—The spatial distribution of dairy cow sheds with the woodland layer analyzed from January 2022 to September 2023 for *C. burnetii* DNA in milk samples (the original data were obtained within the present study). Forest layer was obtained from the Latvia State Forests Geospatial Information service. The circles in blue indicate locations of sheds where all samples analyzed were negative. The circles in red indicate locations of sheds where at least one sample was positive. Scale 1:1,660,000.

In the period from 2012 to 2015, Q fever was more concentrated in the Zemgale region and detected in the Vidzeme region, also near the city Preili (Figure 5A). In the period from 2016 to 2018, Q fever became more prevalent around nearby farms; an example can be observed around the Daugavpils region (Figure 5B). In the period from 2019 to 2021, Q fever was spread all over Latvia, and for the first time, it was detected in Mazsalaca, Aglona, Ludza, Priekuļi, Tērvete and Kocēni counties (Figure 5C).

The findings for the period of 2022–2023 indicated a slight decrease in the prevalence of *C. burnetii* in combined milk and bulk milk samples (Table 1). Despite this, the bacterium remains common in more than half of Latvia’s counties (Figure 5D), underscoring the need for effective control measures.

### 3.4. Determination of Risk Factors

The highest correlation among the woodland data (Figure 5D) was observed between the proportion of forests and the proportion of forest lands (r = 0.948). The proportion of ditches and the proportion of roads were also correlated (r = 0.746) (Table 2). Next, the effect of each individual variable on the probability of Q fever was assessed (Table 3). From all analyzed variables, only the cattle density had a statistically significant impact on Q-fever probability (*p* = 0.002). With an increase in cattle density of 1 unit per km^2^, the probability of Q fever increased by 1.18 times (odds ratio).

Next, the effect of cattle density together with each of the other variables on the probability of Q fever was assessed (Table 4). In Model 1 the effect of the proportion of wetlands on the probability of Q fever was not statistically significant (*p* = 0.097), whereas the effect of cattle density remained statistically significant (*p* = 0.001). Highest counts of positive samples were observed in high-density dairy regions of Zemgale (Saldus, Tukums, Dobele) and parts of Latgale (Preiļi and Jēkabpils), indicating geographic clustering of Q-fever occurrence across the country (Figure 4).

In Model 2 the effect of the proportion of ditches on the probability of Q fever was statistically significant (*p* = 0.016), and the effect of cattle density also remained statistically significant (*p* < 0.001). An increase of 1 percentage point in the proportion of ditches increased the probability of Q fever by 7.69 times (odds ratio). An increase in cattle density of 1 unit per km^2^ increased the probability of Q fever by 1.24 times (odds ratio).

In Model 3 the effect of the proportion of roads on the probability of Q fever was not statistically significant (*p* = 0.135), whereas the effect of cattle density remained statistically significant (*p* = 0.001).

In Model 4 the effect of the proportion of forest on the probability of Q fever was statistically significant (*p* = 0.021), and the effect of cattle density remained statistically significant (*p* < 0.001). An increase of 1 percentage point in the proportion of forest decreased the probability of Q fever by 1.08 times (odds ratio). An increase in cattle density of 1 unit per km^2^ increased the probability of Q fever by 1.27 times (odds ratio).

In Model 5 the effect of the proportion of forest meadow density on the probability of Q fever was not statistically significant (*p* = 0.354), whereas the effect of cattle density remained statistically significant (*p* < 0.001).

In Model 6 the effect of the proportion of overflowing plains on the probability of Q fever was statistically significant (*p* = 0.040), and the effect of cattle density remained statistically significant (*p* < 0.001). An increase of 1 percentage point in the proportion of overflowing plains increased the probability of Q fever by 29.18 times (odds ratio). It is important to note that the confidence interval for the odds ratio ranged from 1.14 to 732.07, indicating substantial uncertainty. An increase in cattle density of 1 unit per km^2^ increased the probability of Q fever by 1.21 times (odds ratio).

## 4. Discussion

To summarize, in the period of 2022–2023, woodlands correlated negatively with the spread of Q fever: regions with more forests had a lower probability of Q fever but regions with fewer forest stands had a higher probability of Q fever (Figure 4, Table 3 and Table 4). The most statistically significant factor, of course, was the density of cattle in the county.

From the retrospective data comparison, it was evident that since no special preventive measures have been taken to control Q fever at the national level, Q fever has spread to over almost all counties of Latvia, with the exception of the six regions around the capital city, Riga. Effective surveillance within control programs generally encompasses case detection, reporting of suspected cases, follow-up investigations, and laboratory confirmation. When *C. burnetii* is widespread and affects the majority of farms, achieving eradication is likely to be impractical. It has been assumed that Q fever is endemic in almost all member states of the European Union [33].

Environmental studies in other countries show that *C. burnetii* DNA concentrations decline sharply with increasing vegetative cover, consistent with the physical trapping of dust, and drier soils are more prone to wind erosion and are also correlated with lower vegetation densities. Overflowing plains have more vegetation. Areas with high groundwater tables or frequent inundation (overflowing plains) tend to have wetter soils, which suppress dust generation. Wet or saturated soils are less prone to wind erosion, so fewer contaminated particles become airborne. Persistent soil moisture prevents the formation of fine, easily entrained dust layers that could harbor *C. burnetii* [20]. The protective effect of vegetation depends on the vegetation type, density, continuity, and scale (a thin hedge is less protective than a continuous forest belt). Most studies reported correlations and modeled effects; very few controlled intervention trials have quantified exact reduction percentages for real farms [25]. Atmospheric dispersion models confirm that rough surface elements, such as forests, shorten plume travel distances and diminish peak concentrations at downwind receptors [23]. Forested wetlands not only trap particulates but also maintain high soil moisture, limiting aerosol generation and transport [33]. The implication for new farms is to avoid locating intensive ruminant operations on open, dry plains. At favorable sites with natural vegetative buffers or higher soil moisture, planting shelterbelts or reforesting windward areas around existing farms can reduce downwind exposure zones. Both a higher forest density and wetter (overflowing) plain conditions act to curtail the windborne dispersal of *C. burnetii*, thereby lowering the risk of Q-fever outbreaks downwind of infected livestock sources [34]. Other studies show that vegetation density and habitat characteristics can significantly affect the presence and behavior of disease vectors. For instance, in regions with uneven surfaces and dense vegetation, such as forests, the distribution of malaria vectors is influenced by factors like humidity and temperature, which can create hotspots for transmission [35,36]. A study on the dispersal patterns of *Phytophthora cinnamomi* in a Spanish heathland found that disease spread was significantly influenced by topography, with flatter areas exhibiting more rapid disease expansion compared to slopes [37]. Moreover, a study on forest health in the United States revealed that denser forests are more susceptible to damage from insects and pathogens, suggesting that while vegetation can provide some protective benefits, it can also create conditions that favor disease outbreaks [38]. Landscape heterogeneity is a major component governing the ecological and evolutionary dynamics of infectious disease. Therefore, while uneven forest surfaces may theoretically lower disease distribution by limiting movement, the actual impact is complex and can vary based on specific ecological contexts and pathogen dynamics [39].

The prevalence of *C. burnetii* in dairy herds and its implications for public health have been subjects of concern not only in Latvia but also across the globe, and incorporating a comparative analysis with other countries provides a broader context for understanding the dynamics of Q-fever transmission and control measures globally. In comparison to the prevalence in Estonia (27.16%) [8], Poland (24.81%) [40], Spain (60.1%) [41], Italy (14.3%) [42] and the global average (37.0%) of the Rabaz study [43], a 15.46% prevalence rate of *C. burnetii* DNA in milk samples from dairy herds in the present study falls within the lower to mid-range of the global prevalence rates reported. However, it is important to note that direct comparisons between studies can be challenging due to differences in sampling methods, diagnostic tests, and the specific populations studied.

The present study was subject to several potential sources of bias. Selection bias could arise if herds included in the analysis were not fully representative of the wider population; to mitigate this, herds were selected based on standardized testing data covering multiple regions. Information bias was possible due to misclassification of herd infection status or inaccuracies in livestock density and land-use data. To reduce this, updated databases were used. Confounding by unmeasured variables, such as herd management practices, wildlife reservoirs, or local climatic conditions, may have influenced the observed associations. Multivariable regression models including cattle density and environmental factors were applied to partially control for these effects. Collinearity among environmental variables and potential spatial autocorrelation between nearby herds were assessed through correlation analyses and model diagnostics. Despite these measures, limitations remain, including the cross-sectional design, which precludes causal inference, wide confidence intervals for certain effect estimates indicating uncertainty, and aggregation of data at the municipality level, which may obscure local variation in Q-fever prevalence. Collectively, these considerations highlight the need to interpret the findings with caution, particularly when extrapolating beyond the study regions.

The actual prevalence data of *C. burnetii* in Latvia’s dairy herds provides valuable insight into the situation within the country and contributes to the broader understanding of Q fever’s global epidemiology. While vegetated areas can provide habitats that support vectors, the specific impact on disease distribution is complex and influenced by various ecological factors. The persistence of the bacterium in a significant proportion of herds highlights the importance of ongoing surveillance, research, and customized control strategies.

## 5. Conclusions

Q fever remains endemic in Latvian dairy cattle herds, with a 15.85% prevalence in the period of 2022–2023. Cattle density was identified as the most significant risk factor (*p* = 0.002), while woodland density showed a weak protective effect (*p* = 0.484). These findings highlight the need for targeted surveillance and control measures to be implemented and to incorporate environmental management to mitigate Q-fever spread. Although potential biases, such as herd selection, measurement errors, and unmeasured confounders, were addressed through careful data sourcing and multivariable analyses, the cross-sectional design and aggregated data impose limitations on causal inference and local-level precision. Nonetheless, our surveillance data and observed historical temporal–spatial changes in disease distribution enhance our understanding of Q-fever dynamics, emphasizing the interplay between livestock density, landscape features (open vs. wooded terrain), and airborne disease transmission. A high cattle density on open, wind-exposed land facilitates aerosolized dispersal and increases prevalence, whereas wooded buffers can serve as natural barriers that limit *C. burnetii* transmission.

## Figures and Tables

**Table 1:** Comparative analysis of the seroprevalence and *C. burnetii* DNA shedding in milk samples from the current study with the results of previous studies.

Time Period [Reference]	Total Number of Samples	Percentage of Positive Samples, % (CI—95% Confidence Interval)	Total Number of Investigated Sheds (Farm, Holding, Herd)	Percentage of Tested Sheds from all Sheds in the Country, %	No. of Positive Sheds/Percentage of Positive Sheds from Tested Sheds, %
Seroprevalence					
2012–2015 [28]	2088	13.40 (9.90–16.90)	1010 (5.00%)	5.00	13.2
2016–2018 (present study)	2016	13.29 (11.88–14.85)	737	6.24	9.77
2019–2021 [27]	1557	20.62 (18.68–22.70)	573	4.85	12.74
*C. burnetii* DNA shedding in milk samples					
2015 [28]	346	10.70 (7.20–14.20)	252	5.00	10.70
2019–2021 [27]	1505	18.34 (16.47–20.37)	1062	12.75	11.02
2022–2023 (present study)	1061	15.85 (13.78–18.17)	657	5.11	10.05

**Table 2:** Correlation among explanatory variables of proportion of forest lands, including swamps, ditches, roads, woodlands, forests, forest meadows and overflowing plains, and cattle density estimated per area of local regions.

	Proportion of Swamps	Proportion of Ditches	Proportion of Roads	Proportion of Woodlands	Proportion of Forests	Proportion of Forest Meadows	Proportion of Overflowing Plains	Cattle Density
Proportion of swamps	-							
Proportion of ditches	−0.14	-						
Proportion of roads	−0.43 *	0.75 *	-					
Proportion of woodlands	−0.03	0.59 *	0.55 *	-				
Proportion of forests	−0.34	0.57 *	0.63 *	0.95 *	-			
Proportion of forest meadows	−0.08	0.09	0.42 *	0.36 *	0.35	-		
Proportion of overflowing plains	−0.42 *	−0.05	0.40 *	−0.12	0.23	0.53 *	-	
Cattle density	0.31	−0.20	−0.29 *	−0.29	−0.37 *	−0.1	0.05	-

* indicates statistically significant correlations, *p* < 0.05.

**Table 3:** The effect of each individual variable (separate GLM models) on the probability of Q fever estimated per area of local regions.

Predictors	Odds Ratios	Confidence Interval	Statistic	*p*
Proportion of swamps	0.81	0.59–1.03	−1.42	0.154
Proportion of ditches	2.67	0.64–11.33	1.34	0.179
Proportion of roads	1.47	0.03–61.15	0.20	0.840
Proportion of woodlands	1.02	0.96–1.08	0.70	0.484
Proportion of forest meadows	0.02	0.00–3.10	−1.50	0.134
Proportion of overflowing plains	13.25	0.57–289.75	1.63	0.103
Cattle density	1.18	1.06–1.30	3.12	0.002 *

* indicates statistically significant value, *p* < 0.05.

**Table 4:** The effect of cattle density together with each of the other variables on the probability of Q fever estimated per area of local regions.

Predictors	Odds Ratios	Confidence Interval	Statistic	*p*
Model 1—Cattle density + Proportion of swamps				
Intercept	0.04	0.02–0.10	−7.41	<0.001 *
Cattle density	1.20	1.07–1.32	3.36	0.001 *
Proportion of swamps	0.79	0.56–0.99	−1.66	0.097
Model 2—Cattle density + Proportion of ditches				
Intercept	0.01	0.00–0.04	−6.65	<0.001 *
Cattle density	1.24	1.10–1.38	3.79	<0.001 *
Proportion of ditches	7.69	1.51–41.77	2.41	0.016 *
Model 3—Cattle density + Proportion of roads				
Intercept	0.01	0.00–0.06	−6.00	<0.001 *
Cattle density	1.22	1.09–1.37	3.43	0.001 *
Proportion of roads	23.45	0.37–1487.06	1.49	0.135
Model 4—Cattle density + Proportion of woodlands				
Intercept	0.00	0.00–0.03	−4.98	<0.001 *
Cattle density	1.27	1.12–1.44	3.83	<0.001 *
Proportion of woodlands	1.08	1.01–1.16	2.31	0.021 *
Model 5—Cattle density + Proportion of forest meadows				
Intercept	0.06	0.01–0.24	−3.85	<0.001 *
Cattle density	1.16	1.04–1.29	2.81	0.005 *
Proportion of forest meadows	0.08	0.00–17.22	−0.93	0.354
Model 6—Cattle density + Proportion of overflowing plains				
Intercept	0.02	0.00–0.05	−7.11	<0.001 *
Cattle density	1.21	1.08–1.35	3.36	0.001 *
Proportion of overflowing plains	29.18	1.14–732.07	2.05	0.040 *

* indicates statistically significant value, *p* < 0.05.

## Data Availability

The original contributions presented in this study are included in the article. Further inquiries can be directed to the corresponding author.
